# Exposure to selected limonene oxidation products: 4-OPA, IPOH, 4-AMCH induces oxidative stress and inflammation in human lung epithelial cell lines

**DOI:** 10.1016/j.chemosphere.2017.10.065

**Published:** 2018-01

**Authors:** Dorelia Lipsa, Josefa Barrero-Moreno, Mehmet Coelhan

**Affiliations:** a Technische Universität München, Research Center Weihenstephan for Brewing and Food Quality, Alte Akademie 3, Freising-Weihenstephan, Germany; b European Commission, DG Joint Research Centre, Ispra, Italy

**Keywords:** Limonene oxidation products, Reactive oxygen species, Glutathione, Inflammation, Human pulmonary cells

## Abstract

Limonene oxidation products (LOPs) have gained interest on their harmful health effects over time. Recently, studies have shown that the selected LOPs: 4-oxopentanal (4-OPA), 3-isopropenyl-6-oxo-heptanal (IPOH) and 4-acetyl-1-methylcyclohexene (4-AMCH) have sensory irritation effects in mice and inflammatory effects in human lung cells. This study was therefore undertaken to investigate the potential capacity of 4-OPA, IPOH and 4-AMCH to cause cell membrane damage, oxidative stress and inflammation in human bronchial (16HBE14o-) and alveolar (A549) epithelial cell lines.

Overall results suggest that 4-OPA, IPOH have cytotoxic effects on human lung cells that might be mediated by ROS: the highest concentration applied of IPOH [500 μM] enhanced ROS generation by 100-fold ± 7.7 (A549) and 230-fold ± 19.9 (16HBE14o-) compared to the baseline. 4-OPA [500 μM] increased ROS levels by 1.4-fold ± 0.3 (A549) and by 127-fold ± 10.5 (16HBE14o-), while treatment with 4-AMCH [500 μM] led to 0.9-fold ± 0.2 (A549) and 49-fold ± 12.8 (16HBE14o-) increase. IPOH [500 μM] caused a decrease in the thiol-state balance (e.g. after 2 h, GSH:GSSG was reduced by 37% compared to the untreated 16HBE14o-cells). 4-OPA [500 μM] decreased the GSH:GSSG by 1.3-fold change in A549 cells and 1.4-fold change in 16HBE14o-cells. No statistically significant decrease in the GSH:GSSG in A549 and 16HBE14o-cell lines was observed for 4-AMCH [500 μM]. In addition, IPOH and 4-OPA [31.2 μM] increased the amount of the inflammatory markers: RANTES, VEGF and EGF. On the other hand, 4-AMCH [31.2 μM] did not show inflammatory effects in A549 or 16HBE14o-cells.

The 4-OPA, IPOH and 4-AMCH treatment concentration and time-dependently induce oxidative stress and/or alteration of inflammatory markers on human bronchial and alveolar cell lines.

## Highlights

• 4-OPA, IPOH and 4-AMCH were tested on human pulmonary cells.
• Markers of cell death, oxidative stress and inflammation investigated in lung cells.
• Non-cytotoxic concentrations of IPOH and 4-OPA induced intracellular ROS accumulation.
• Alteration in the inflammatory markers were observed after treatment with IPOH and 4-OPA.
• 4-AMCH caused less toxic effects compared to 4-OPA and IPOH.

## Graphical abstract

**Figure:**
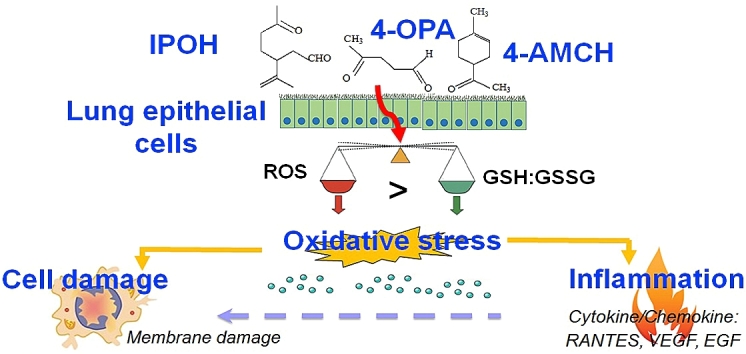

## 1 Introduction

**Fig. 1:**
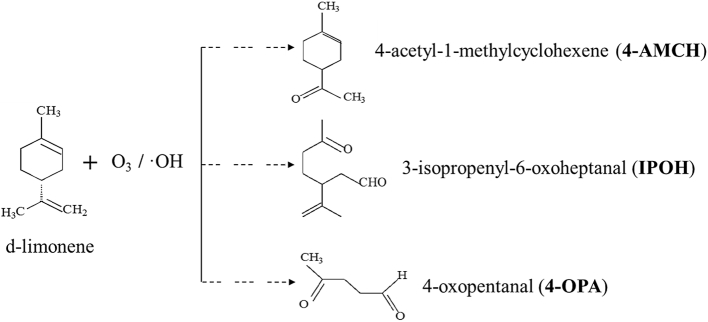
Schematic representation of limonene reaction with O_3_, OH leading to the formation of carbonyl species: 4-OPA, IPOH and 4-AMCH.

Environmental chemical exposure has been linked to a wide range of human health hazards such as neurodegenerative diseases, endocrine disorders, cardiovascular disease and cancer (Pope et al., 2004, Uzoigwe et al., 2013). Terpenes are the greatest part of volatile organic compounds present in living surroundings and are widely used as constituents in household products, particularly in cleaning and personal care products (Sarwar et al., 2004, Steinemann, 2009). Their reaction with indoor and outdoor ozone (O_3_) as well as with hydroxyl radicals (OH) leads to various oxidation products (Calogirou et al., 1999). Some of these terpene oxidation products (TOPs) can act as carcinogens, asthma promoters and irritants (Wolkoff et al., 2006). Although previous studies have provided information about the health effects of certain TOPs (e.g. formaldehyde, hydroperoxides), little attention has been given to the potential health risks of the carbonyl species formed: 4-oxopentanal – 4-OPA, 3-isopropenyl-6-oxo-heptanal – IPOH, 4-acetyl-1-methylcyclohexene – 4-AMCH (Fig. 1).

One of the carbonyl species selected for this study, 4-OPA is a typical key oxidation reaction product of limonene (Rossignol et al., 2012), geraniol (Forester et al., 2007), alpha-terpineol (Wells, 2005) – fragrances that are used in a number of consumer and household products (Nazaroff and Weschler, 2004). Squalene - present in the outermost layer of the skin - is another important source of 4-OPA (Fruekilde et al., 1998). Besides 4-OPA, both IPOH and 4-AMCH were detected after the use of cleaning products containing terpenes (e.g. limonene) in a test house and a climate chamber (Nørgaard et al., 2014, Rossignol et al., 2013).

To date, few studies are characterizing the potential toxicity of 4-OPA, IPOH and 4-AMCH. Moreover, it is not clear if the presence and/or formation of this chemical group might be one of the contributors to the toxic health effects of limonene-ozone mixtures reported in many studies (Sunil et al., 2007, Wolkoff et al., 2008, Anderson et al., 2013). Among the few toxicological studies published to date that have investigated the potency of 4-OPA, IPOH and 4-AMCH, results have showed:

(A) *in vivo* (1) by using a mouse bioassay, a relatively high estimated sensory irritation potency was noticed for the selected chemicals as follows: IPOH with no observed (adverse) effect at a level (NO(A)EL) of around 1.6 ppmv; 4-AMCH with a NOEL value of around 13 ppmv while 4-OPA with an estimate for sensory irritation of around 3.4 ppmv (Wolkoff et al., 2013); (2) mice exposed to 4-OPA, through both dermal and pulmonary routes of exposure, showed that 4-OPA can be an irritant (e.g. at 1.97 mM 4-OPA, p < 0.01) and a sensitizer (e.g. 0.02 mM, p < 0.01). At a concentration of 0.08 mM, 4-OPA increased airway responsiveness, caused neutrophil and lymphocytes influx (Anderson et al., 2012);

(B) *in vitro* (1) by exposing the pulmonary epithelial cells (A549) to a gas phase containing 65 ppm 4-OPA, inflammatory markers levels of IL-8 and TNF-alpha were increased after exposure (8, 12, 24 h), while IL-6 and GM-CSF were significantly augmented at 12 h (e.g. 1059 pg mL^−1^ for IL-6 and 17 pg mL^−1^ for GM-CSF) (Anderson et al., 2010); (2) recent *in vitro* findings have shown an increase of some inflammatory markers [e.g. interleukin-6 (IL-6), tumour necrosis factor alpha (TNF-alpha)] when human alveolar (A549) and bronchial (16HBE14o-) epithelial cell lines were exposed to 4-OPA, IPOH and 4-AMCH at concentrations of up to 50 μM. In the case of IPOH, a concentration of 1.5 μM stimulated the release of IL-6, IL-8 and TNF-alpha in bronchial cells (3.0-, 2.3-, 1.4-fold change respectively compared to untreated cells). Under the same experimental conditions, 4-OPA induced a marked increase of IL-8 (2.4-fold), IL-6 (3.3-fold) and TNF-alpha (2.2-fold). In comparison, bronchial cells exposed to 4-AMCH showed a 2-fold change in (IL-8), a 2.5-fold change in (IL-6) and a 1.0-fold change in (TNF-alpha) (Lipsa et al., 2016).

Previous investigations carried out on lysosomal integrity by Neutral red uptake assay (NRU) using both A549 and 16HBE14o-cell lines exposed to 4-OPA (0.2–115 mM), IPOH (0.03–17.5 mM) and 4-AMCH (0.01–5.8 mM), have shown that the cellular viability was reduced much more by 4-OPA [IC_50_ = 1.6 mM (A549) and 1.45 mM (16HBE14o-)] compared to IPOH [IC_50_ = 3.5 mM (A549) and 3.4 mM (16HBE14o-)] and 4-AMCH [IC_50_ could not be calculated] (Lipsa et al., 2016).

Evidence has shown that inflammatory cytokines/chemokines (e.g. IL-6 and TNF-alpha) could promote reactive oxygen species (ROS) generation (Wang et al., 2014). Inflammatory lung diseases could be induced by the imbalance between the generation and removal of ROS, a phenomenon known as oxidative stress (Dalleau et al., 2013, Klaunig et al., 2010).

ROS refers to a number of entities (e.g. free radicals) which are difficult to be detected *in vivo* (Halliwell, 2006). On one hand, ROS are needed in various biological functions such as cell growth and differentiation (Assim and Reem, 2012). On the other hand, excessive production of ROS and reactive nitrogen species (RNS) might induce cell damage leading to cell death (e.g. apoptosis) (Dixon and Stockwell, 2014) or they might lead to progressive inflammatory diseases (e.g. asthma, etc.) (Rahman and MacNee, 2000, Rossignol et al., 2013). Recent evidence has shown that ROS initiates the production of inflammatory cytokines/chemokines such as Regulated on Activation Normal T-cells Expressed and Secreted (RANTES), Vascular Endothelial Growth Factor (VEGF), interleukin-8 (IL-8), interleukin-6 (IL-6), interleukin-10 (IL-10), monocyte chemoattractant protein-1 (MCP-1) and those being induced via redox-dependent signalling pathways (Sozzani et al., 2005, Ushio-Fukai, 2007, Fay et al., 2006). RANTES has been linked to allergic inflammation of asthma (Zietkowski et al., 2008) and VEGF has been associated with the high metastatic potential of non-small cell lung cancers (Lee et al., 2016. The epidermal growth factor (EGF) is a potent stimulant of human airway smooth muscle proliferation (Hirst et al., 1992) and an increase in its expression has been reported in the patients with chronic bronchitis (Vignola et al., 1997). If inflammation is left unresolved, this might induce permanent structural damage of the airways (e.g. cystic fibrosis).

To protect or defend against the increased ROS/reactive nitrogen species (RNS) levels, cells have an antioxidant defence system (e.g. superoxide dismutase – SOD, glutathione – GSH) (Poljsak et al., 2013). Glutathione (GSH) is a tripeptide able to defend the cells against the action of contaminants/carcinogens by reacting with electrophilic or oxidizing species. Upon oxidation GSH is transformed to glutathione disulfide (GSSG) where GSH traps any nitric oxide or peroxynitrite (Carmel-Harel and Storz, 2000). The decrease of GSH amount, so modification of the GSH:GSSG ratio is an indicator of oxidative stress (Anderson, 1998). Under normal conditions, the optimal GSH:GSSG ratio exceeds 100, while in cases of oxidative stress the GSH:GSSG ratio is significantly lower (e.g. between 10 and 1) (Hassan and Fridovich, 1980). Alterations in the ratios of glutathione have been related to diseases such as asthma, bronchitis, pulmonary fibrosis etc. (Biswas and Rahman, 2009).

Because the first signs of a potential toxic effect occur at the cellular level, the current work was designed to assess the contribution of IPOH, 4-OPA and 4-AMCH, to induce toxic effects in human bronchial (16HBE14o-) and alveolar (A549) epithelial cell lines. The choice of 16HBE14o- and A549 cell lines is subject to the considerations: (1) 16HBE14o-serve as barrier protection of the lung against airborne compounds (Heijink et al., 2010), (2) both selected cell lines are important modulators of immune responses (3) the A549 cell line has been commonly used as an *in vitro* model for a type II pulmonary epithelial cells (Lieber et al., 1976).

The present *in vitro* study was undertaken (1) to determine the ROS production in the selected lung cell lines as a response to their exposure to the above-mentioned carbonyls as determined by the 2′,7′-dichlorofluorescin diacetate (DCFH-DA) assay (2) to measure the cellular antioxidant capacity (alteration of glutathione level species: GSH, GSSG) by applying a high-performance liquid chromatography (HPLC) method with ultraviolet (UV) detection and (3) to further assess the potential inflammatory capacity of 4-OPA, IPOH and 4-AMCH, the following cytokine/chemokines: VEGF, RANTES and EGF were determined with enzyme-linked immunosorbent assays (ELISA).

## 2 Materials and methods

### 2.1 Chemical reagents and materials

Trifluoroacetic acid (TFA), picric acid, sodium perchlorate, 2′,7′-dichlorofluorescein diacetate (DCFH-DA), metaphosphoric acid (MPA), *tert*-Butyl hydroperoxide (TBHP), 4-oxopentanal (4-OPA), acetonitrile, 3-isopropenyl-6-oxoheptanal (IPOH), 4-acetyl-1-methylcyclohexene (4-AMCH), sodium dodecyl sulfate, ammonium sulfate, glutathione (GSH), glutathione disulphide (GSSG) standards were obtained from Sigma Aldrich (Sigma Aldrich, St. Louis MO, USA).

### 2.2 Biological reagents and materials

Minimum Essential Medium (MEM), fetal bovine serum (FBS), penicillin-streptomycin, 0.25% trypsin/EDTA, phosphate buffered saline (PBS), Roswell Park Memorial Institute medium (RPMI 1640) and 4-2-hydroxyethyl-1-piperazinyl-ethanesolfonic acid (HEPES) were procured from Invitrogen (USA). The mammalian protein extraction reagent (M-PER) was obtained from Thermo Fischer (Italy).

The human lung epithelial carcinoma cell line (A549) was bought from American Type Culture Collection (ATCC #: CCL-185). The human bronchial epithelial cell line (16HBE14o-) was delivered by Dr. Dieter C. Gruenert (Cardiovascular Research Institute at the University of California, San Francisco, Calif., USA) for research purposes.

### 2.3 Analytical methods used

#### 2.3.1 The GC-FID method conditions to assess the chemical stability of 4-OPA, 4-AMCH, IPOH dissolved in the culture medium

The theoretical concentration of the test substance may be modified by the medium composition (e.g. serum proteins may bind the test substances thus the effective concentration may differ) (Berg et al., 2002). Therefore, the stability of chemicals used to prepare the culture medium was analysed following the GC-FID method described elsewhere (Lipsa et al., 2016).

#### 2.3.2 The HPLC-UV method conditions to detect and quantify GSH, GSSG simultaneously in human pulmonary cell lines

The simultaneous determination of intracellular glutathione (GSH, GSSG) molecules was carried out following the HPLC-UV method described elsewhere (Lipsa et al., 2015). The proteins were quantified by Bradford method following the manufacturer's protocol.

The peak area of the glutathione standards was plotted on a regression linear curve (concentration vs. area). The peak area of the samples was normalized based on the protein content of each biological sample.

### 2.4 In-vitro methods used

#### 2.4.1 Maintenance of the cell cultures

The A549 cell line was maintained in RPMI 1640 medium supplemented with 10% FBS and penicillin 100 U mL^−1^ and streptomycin 100 μg mL^−1^; the 16HBE14o-cell line was grown in MEM medium enriched with 10% FBS, 1% l-glutamine and antibiotics.

#### 2.4.2 Cellular viability – damage of cell membrane measured by lactate dehydrogenase (LDH) assay

Loss of membrane integrity was assessed by quantifying the lactate dehydrogenase (LDH) released into the cellular medium. The quantity of LDH released was determined following the manufacturer's instructions (Cytotoxicity detection Kit (LDH), Roche Molecular Biochemicals, Lewes, UK).

Briefly, after chemical treatment, aliquots of culture media and the reagent mixture provided by the kit were mixed in a 96-well plate followed by 30 min incubation at room temperature in the dark. Stop solution was added and the plate was read at 490 nm.

Background subtraction was applied for all the measurements, thereafter the obtained measurements of the absorbance - the optical densities (ODs) - were normalized by dividing the OD of each well by the average OD of the negative control (cells untreated).

#### 2.4.3 Oxidative stress - reactive oxygen species assessed by 2′, 7′-dichlorofluorescein diacetate assay

The intracellular generation of ROS was determined using a cell permeant reagent 2′, 7′-dichlorofluorescein diacetate (DCFH-DA). This can passively enter the cells and be deacetylated by cellular esterase to a non-fluorescent compound (DCFH). Later DCFH can be oxidized by ROS into a highly fluorescent compound, 2′, and 7’ –dichlorofluorescin (DCF) (Halliwell and Whitemann, 2004).

In brief, 1 × 10^5^ A549 and 3 × 10^5^ 16HBE14o-cells were plated per well in 96-well black plates. Subsequently, cells were washed once with PBS and stained with DCFDA 25 μM for 45 min at 37 °C. Then, cells were incubated with 200 μl/well target chemicals at different concentrations (dilution factor of 4, minimum concentration 0.002 to maximum of 500 μM) for different time intervals (60, 120, 210 and 1440 min). After the exposure time had passed, the cells were washed twice with PBS. The fluorescent plate reader was set at an excitation wavelength of 485 nm, and emission wavelength 535 nm.

Cell negative control (e.g. culture medium without chemicals) and cell positive control (e.g. 55 μM of *tert*-Butyl hydroperoxide) were also considered.

The background controls were subtracted from the measured fluorescence values. Results were expressed as the mean ± SD of experiments with eight replicates.

#### 2.4.4 Inflammatory response assessed by cytokine/chemokine assay

The release of cytokines/chemokines was quantified in the cell culture supernatants using a MILLIPLEX MAP Kit (HCYTOMAG-60 K, Millipore, Billerica, MA). The simultaneous measurement of the following human cytokines/chemokines: RANTES, VEGF and EGF was carried out following the manufacturer's protocol. The plate was scanned immediately on a Luminex^®^ 100™/200™ platform (Luminex Corporation) with xPONENT 3.1 software.

In addition to the negative and positive controls, target chemicals, which were solubilized in the cellular medium were included in the plate to overcome any potential chemical interference with the assay.

The background controls were subtracted from the measured fluorescence values and the cytokine/chemokines quantification in the samples was done with a 5-parameter logistic curve. Final concentrations were determine from the mean fluorescence intensity and expressed in pg mL^−1^.

### 2.5 Statistical analyses

All assays were performed in at least three independent experiments, where in every experiment at least three biological replicates were used all from the same cell batch. The triplicate and the statistical significance were determined using ANOVA with unpaired *t*-test. Each treatment group was compared to cells untreated (negative control). A difference was considered to be statistically significant when p < 0.05.

## 3 Results

### 3.1 Effects of 4-OPA, IPOH and 4-AMCH on membrane integrity

**Fig. 2:**
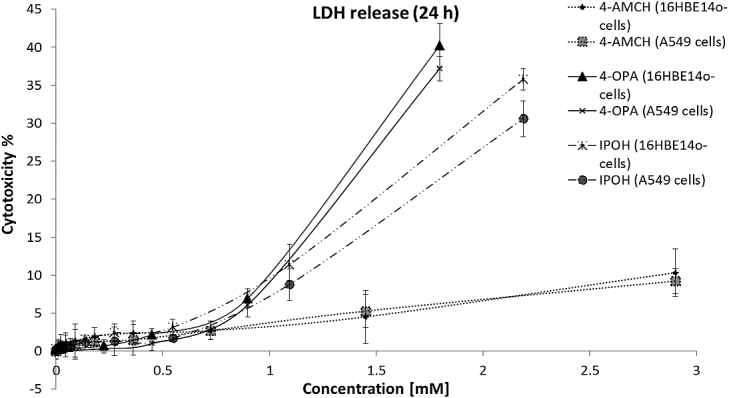
Cell membrane damage assessed by LDH assay in A549 and 16HBE14o-cells after 24-h exposure to the tested limonene-ozone reaction products: 4-OPA, IPOH and 4-AMCH. Data is expressed as the means of the three independent experiments.

To determine the harmful effects of 4-OPA, IPOH and 4-AMCH on A549 and 16HBE14o-cells, cell viability was evaluated based on membrane integrity using the lactate dehydrogenase (LDH) assay. Fig. 2 shows the results of the LDH assay performed for both cell lines incubated for 24 h with the IC_50_ concentrations of each chemical, determined in our previous work: 4-OPA [IC_50_ = 1.6 mM (A549) and 1.45 mM (16HBE14o-)]; IPOH [IC_50_ = 3.5 mM (A549) and 3.4 mM (16HBE14o-)]. Since the IC_50_ of 4-AMCH could not be calculated (Lipsa et al., 2016), the concentration used was half of its maximum soluble concentration: 2.9 mM.

In both cell types, 4-OPA and IPOH induced a significant increase in LDH release starting from 1 mM (p < 0.05). The amount of LDH released increased in a concentration-dependent manner by 35.2% (p < 0.001) after exposure to 4-OPA [1.8 mM]. In particular, IPOH [1.1 mM] induced 8.8% (p < 0.05) of cytotoxicity in A549 cells and 11.4% (p < 0.05) in 16HBE14o-cells. At a higher concentration, IPOH [2.2 mM] increased the LDH release to 30.6% (p < 0.01) in A549 and 35.5% (p < 0.001) in 16HBE14o-. At a lower concentration, IPOH [0.6 mM] did not significantly change the amount of LDH released compared to the control cells. Cells incubated with 4-AMCH [0.7 mM] did not show any increase in LDH release compared to the control A549 and 16HBE14o-cells. On the contrary, 4-AMCH [2.9 mM] induced a significant increase in LDH release, 10.3% in 16HBE14o-cells and 9.2% in A549 cells (p < 0.05).

The highest test compound concentration used for the study of oxidative stress and inflammation was determined by the initial cytotoxicity test to be 0.5 mM, below which LDH release was not observed.

### 3.2 Effects of 4-OPA, IPOH and 4-AMCH on reactive oxygen species (ROS) generation

To evaluate whether or not 4-OPA, IPOH and 4-AMCH could induce oxidative stress in alveolar (A549) and bronchial (16HBE14o-) epithelial cells, the measurement of ROS levels was carried out after cells were incubated with ten different concentrations (obtained by dilution factor of 4) ranging from 0.002 to 500 μM for each chemical, for different lengths of time (1, 2, 3.5 and 24 h).

Results showed that at the lower concentrations (0.002–8 μM), the fluorescent signal of both cell lines was not significantly different when compared to the untreated cells. This tendency was seen for all tested compounds (data not shown). Therefore, those fluorescent signals were all pooled as an estimation of the background signal (S0) and further treatment was done for the three highest concentrations (31.2, 125 and 500 μM) based upon (S-S0).

**Fig. 3:**
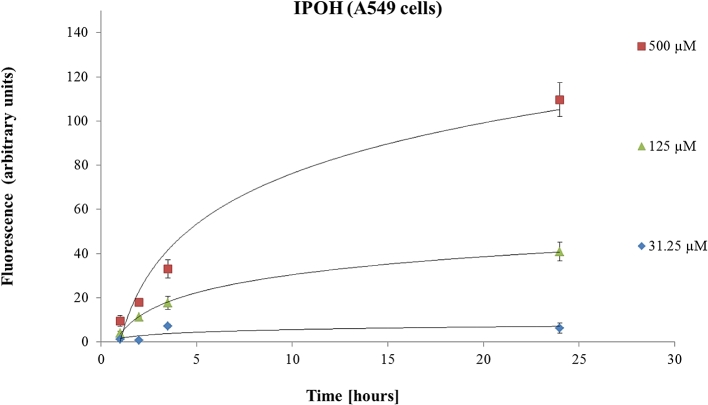
The effects of IPOH on ROS production measured in A549 cell line. Data are presented as the mean ± SD fitted to logarithmic trend lines.

As presented in Fig. 3, A549 cells incubated with IPOH [500 μM] for 24 h, produced a 100-times stronger ROS signal compared to the baseline. Moreover, a concentration-dependent effect of IPOH on ROS formation was noted.

**Fig. 4:**
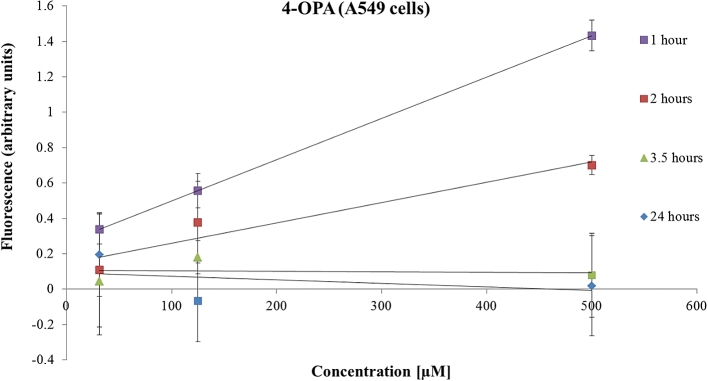
The effects of 4-OPA on ROS production measured in A549 cell line. Data are presented as the mean ± SD.

On the other hand, after 1 h, 4-OPA induced a significant increase in the ROS signal 1.4 times higher (p < 0.05) than the baseline at 500 μM. Curiously, it was noticed that ROS production peaked at 1 h (Fig. 4) and then gradually declined, returning to baseline level within 24 h in A549 cells (1 > 2 > 3.5 > 24 h).

4-AMCH [500 μM] had a similar time behaviour (1 > 2 > 3.5 > 24 h) to 4-OPA [500 μM] regarding the ROS level measured in A549 cells (data not shown).

**Fig. 5:**
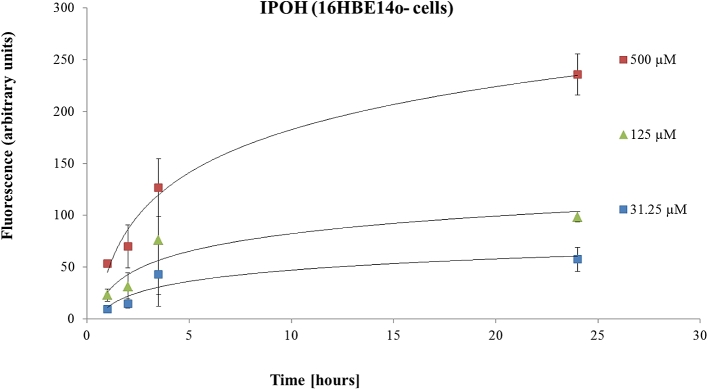
The effects of IPOH tested at different concentrations (31.2, 125 and 500 μM) on ROS production measured in 16HBE14o-cell line. Data are presented as the mean ± SD.

To eliminate the risk that the observed effects of IPOH are specific to A549 cells, experiments were performed considering also the 16HBE14o-cell line. As for the A549 cells, a concentration-dependent effect was observed for the IPOH on ROS generation. As shown in Fig. 5, IPOH [500 μM] led to a dramatic increase in fluorescence intensity, 230 fluorescence units (FU) in 16HBE14o-cells compared to the baseline.

**Fig. 6:**
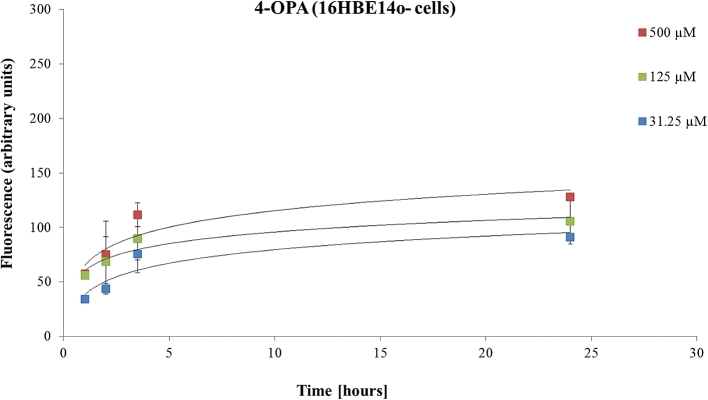
The effects of 4-OPA tested at different concentrations (31.2, 125 and 500 μM) on ROS production measured in a 16HBE14o-cell line. Data are presented as the mean ± SD.

Treatment of 16HBE14o-cells with 4-OPA also resulted in a concentration-dependent increase in the ROS generation, demonstrated by the increase in the intensity of DCF fluorescence (Fig. 6). However, 4-OPA contributed to a lower increase in the ROS amount compared to IPOH (e.g. 127 FU for 4-OPA than 230 FU for IPOH), using the same concentration (500 μM) and time conditions (24 h). On the other hand, treatment with 4-AMCH [500 μM] led to only 49 FU, measured after 24 h.

### 3.3 Effects of 4-OPA, IPOH and 4-AMCH on intracellular reduced (GSH), oxidized (GSSG) glutathione levels

As IPOH, 4-OPA and 4-AMCH exerted oxidative effects by causing excessive ROS accumulation in both bronchial and alveolar cells, it was hypothesized that this would render the cells highly dependent on GSH to preserve their redox balance.

The two cell lines were thus incubated with either the cell culture medium (=negative control) or with the selected chemicals at various concentrations: 1.5, 31.2 and 500 μM. The compound concentrations were chosen based on their ROS formation potency: 500 μM corresponds to the highest ROS levels generated in the cells, 31.2 μM represents the lowest concentration that generated ROS which was statistically different from the ROS levels measured in untreated cells.

The GSH content of A549 cells was 1.3 times that of 16HBE14o-cells (26.3 ± 1.1 nmol mg^−1^ protein).

The intracellular glutathione species: GSH and GSSG were measured in both cell lines at 2 and 24 h post-treatment and their ratio was calculated for every cell line exposed to the selected chemicals at different concentrations (see Table 1). Depletion of the GSH: GSSG ratio was observed consistently for A549 and 16HBE14o-cells after IPOH and 4-OPA exposure at each time point, at the following concentrations: 31.2 and 500 μM. After 2 h, a significant reduction in the GSH: GSSG ratio, by 37%, was observed in the 16HBE14o-cells treated with IPOH [500 μM] compared with the untreated cells (control). IPOH [31.2 μM] induced changes in the GSH: GSSG ratio resulting in a decrease in their ratio of 14.4%. The ratio of the GSH: GSSG was also reduced, by 31.6% more in the A549 cells, after 2 h post treatment, than in the untreated A549 cells.

**Table 1:** Effects of 4-AMCH, IPOH and 4-OPA exposure on intracellular redox state of A549 and 16HBE14o-cells as indicated by the ratio of GSH to GSSG. Mean values ± standard deviations. The results presented here are representative of three biological replicates.

Cell line	Chemicals	Concentration [μM]	GSH:GSSG 2 h	GSH:GSSG 24 h
A549	Untreated cells	0	17.3 ± 0.5	
	4-AMCH	500	16.9 ± 0.3	15.8 ± 0.8
		31.2	17.1 ± 0.9	17.1 ± 0.2
		1.5	17.4 ± 1.1	17.2 ± 0.1
	IPOH	500	11.8 ± 0.7	10.5 ± 0.4
		31.2	14.8 ± 1.3	16.7 ± 0.5
		1.5	17.1 ± 0.6	17.2 ± 0.4
	4-OPA	500	13.4 ± 1.0	11.7 ± 0.4
		31.2	15.3 ± 1.3	17.0 ± 0.1
		1.5	17.1 ± 0.2	17.4 ± 0.7
16HBE14o-	Untreated cells	0	14.6 ± 1.0	
	4-AMCH	500	14.6 ± 0.3	14.6 ± 0.4
		31.2	14.6 ± 0.8	14.6 ± 0.7
		1.5	14.5 ± 1.0	14.5 ± 0.3
	IPOH	500	9.2 ± 1.9	8.1 ± 0.5
		31.2	12.5 ± 1.3	13.6 ± 0.5
		1.5	14.6 ± 0.5	14.5 ± 0.2
	4-OPA	500	10.1 ± 0.1	9.3 ± 1.1
		31.2	13.4 ± 1.3	14.2 ± 0.1
		1.5	14.3 ± 0.4	14.7 ± 0.2

To evaluate whether 4-OPA or 4-AMCH reduces the GSH: GSSG ratio, the effect was tested in both A549 and 16HBE14o-cells. The average ratio of GSH: GSSG in the A549 cells which were treated with 4-OPA [500 μM] was 1.30 times lower compared to the that of the untreated A549 cells. On the other hand, the GSH: GSSG ratio of 16HBE14o-cells treated with 4-OPA [500 μM] was 1.44 times lower than the control, untreated cells.

No significant modifications in the GSH: GSSG ratio were observed in both cell lines treated with 4-OPA, IPOH or 4-AMCH at 1.5 μM.

### 3.4 Effects of 4-OPA, IPOH and 4-AMCH on the markers of inflammation

Given that GSH depletion and ROS overproduction play a significant role in inflammatory response, levels of inflammatory cytokines/chemokines such as RANTES, VEGF and EGF were measured in both A549 and 16HBE14o-cells treated with 4-OPA, IPOH and 4-AMCH at different concentrations.

Thus, to determine whether there is a change in the level of RANTES, VEGF and EGF, both A549 and 16HBE14o-cell lines were treated for 24 h with 4-OPA, IPOH and 4-AMCH [1.5 and 31.2 μM].

Treatment of 16HBE14o-cells with 31.2 μM 4-OPA resulted in a significantly increased (One-way ANOVA; p < 0.05) level of RANTES (1.9-fold change), as well as higher amounts of VEGF (1.5-fold change) and EGF (1.7-fold change). Significantly higher levels of RANTES (2.0-fold change), VEGF (1.4-fold change) and EGF (1.5-fold change) were observed in supernatants of the 16HBE14o-cells exposed to IPOH [31.2 μM]. On the other hand, RANTES, VEGF and EGF were not detected in the supernatants of the bronchial cells (16HBE14o-) when they were exposed to 31.2 μM 4-AMCH (see Table 2).

**Table 2:** The fold changes calculated for the inflammatory markers: RANTES, VEGF and EGF released from A549 and 16HBE14o-cells exposed 4-OPA, IPOH, 4-AMCH at different concentrations [1.5 and 31.2 μM].

Cell lines	Chemical	Conc. [μM]	Cytokines/chemokines (fold changes)		
			RANTES	VEGF	EGF
A549	4-OPA	1.5	1.6	1.4	1.0
		31.2	1.4	1.4	1.6
	IPOH	1.5	1.5	1.4	1.3
		31.2	1.4	1.4	1.5
	4-AMCH	1.5	1.0	1.1	1.0
		31.2	1.1	1.2	1.1
16HBE14o-	4-OPA	1.5	1.8	1.2	1.3
		31.2	1.9	1.5	1.7
	IPOH	1.5	1.8	1.2	1.3
		31.2	2.0	1.4	1.5
	4-AMCH	1.5	1.0	1.1	1.0
		31.2	1.1	1.1	1.0

As illustrated in Table 2, both IPOH and 4-OPA [31.2 μM] enhance the levels of all the inflammatory markers (RANTES, VEGF and EGF) measured in the A549 cells, whereas 4-AMCH did not induce any changes in the amount of inflammatory markers when compared to the untreated cells.

## 4 Discussion

In the present study both oxidative and inflammation effects of the three representative limonene-oxidation reaction products: 4-OPA, IPOH and 4-AMCH were assessed in human alveolar (A549) and bronchial (16HBE14o-) epithelial cell lines. Respiratory epithelial cells are the first line of defense so a deeper knowledge of the potentially harmful impact of chemicals may help us to understand how epithelial cells respond to lung injury (Vareille et al., 2011).

The results of the present work regarding cell membrane damage, measured by LDH release in A549 and 16HBE14o-cells exposed to 4-OPA, IPOH and 4-AMCH, are in agreement with the results obtained by NRU assay in our previous study (Lipsa et al., 2016). Cytotoxic action of the investigated chemicals after 24 h of treatment follows this order: 4-OPA > IPOH > 4-AMCH since 4-AMCH [0.7 mM] did not affect cell viability at all while 4-OPA [1.8 mM] and IPOH [1.1 mM] had higher cytotoxic effects, demonstrated by the increased LDH release. Increases in LDH release are usually detected in necrotic cell injury but may also reveal cell death due to late-apoptosis (Chan et al., 2013). Under some conditions (e.g. IPOH [2.2 mM], 16HBE14o-cells exhibited more LDH release (e.g. 35.5%, p < 0.001) than A549 cells (e.g. 30.6%, p < 0.01) which can be explained by the fact that the cancer A549 alveolar epithelial cell line is more resistant to the toxic effects of the examined chemicals than the normal 16HBE14o-bronchial epithelial cell line.

A higher ROS accumulation was determined in A549 cells treated with IPOH compared to the ROS amount induced by 4-OPA or 4-AMCH. A possible explanation of this difference could be that 4-OPA is less reactive than both IPOH and 4-AMCH and does not have a C=C double bond. On the other hand, the difference in ROS formation between 4-AMCH and IPOH may be due to their chemical structure. IPOH is an open-chain compound (more flexible) while 4-AMCH is a ring compound. This might suggest that IPOH would be able to reach cellular targets more easily than 4-AMCH. Moreover, IPOH is more reactive due to its external C=C double bond than 4-AMCH which has an internal C=C double bond in the ring.

Interestingly, it was found that IPOH [500 μM] induced higher amounts of ROS in 16HBE14o- (2-fold change) than in A549 cells. The difference in ROS levels generation detected in the two cell lines. This may have several causes, such as different esterase activity in the selected cell lines (Schmiedebergs's, 1985), different intracellular localization of fluorochromes (Robinson et al., 1988) or differences in the oxidative metabolism of the cell lines (Ertel et al., 2006). The low esterase activity has direct implications in the detection of ROS since the non-fluorescent reagent DCFH-DA needs to be hydrolyzed to DCFH which is further oxidized to fluorescent dichlorofluorescin (DCF) by the action of free radicals (Curtin et al., 2002).

It has been demonstrated that the esterase activity in tumour lung cells is lower than in normal lung cells, due to the significant biochemical differences. For the selected cell lines of the present study, no specific data were found in the literature, but with other cells it has been shown that when stimulated with phorbol myristate acetate (PMA), monocytes and neutrophils induce a different increase in the fluorescence signal of the two cell lines. The authors suggested that this can happen due to the difference in the oxidative mechanism of the cells (Robinson et al., 1998).

Numerous publications have stated that the intracellular glutathione levels in cancer cells are higher than in normal cells, leading to an augmentation in the resistance of A549 in presence of free radicals (Russo et al., 1986, Traverso et al., 2013). This might explain our observation that 16HBE14o-cells induce higher ROS amounts than A549 cells.

The imbalance between ROS or RNS and their elimination by enzymatic (e.g. superoxide dismutase etc.) or non-enzymatic (e.g. glutathione) antioxidants has been directly linked to cell death, many inflammatory diseases and cancer (Dalleau et al., 2013, Klaunig et al., 2010). In particular, RNS can indirectly induce modifications of proteins which can increase the risk of mutagenesis (Trachootham et al., 2008). Therefore, the antioxidant protection reaction by glutathione was investigated by measuring the changes that occurred in the intracellular glutathione ratios of GSH: GSSG.

The intracellular reduction in glutathione content in the untreated alveolar epithelial cells (A549) of 34.75 ± 0.3 nmol mg^−1^ protein (n = 8) is within the concentration range obtained in other published studies (Spadaro et al., 2011). It was found that the average level of GSH in A549 cells was higher (1.3-fold higher) than those in 16HBE14o-cells. This result is supported by several studies (Calvert et al., 1998, Estrela et al., 2006) and it is based on the concept that in cancer cell lines (e.g. A549), the intracellular reduction in glutathione is at higher levels than in normal cell lines (e.g. 16HBE14o-).

The GSH: GSSG ratio may be used as a potential indicator of oxidative stress (Serru et al., 2001, Owen and Butterfield, 2010, Ahner et al., 2002). Through extensive studies on the GSH: GSSG ratio steady state level, it was found that a decrease in the GSH: GSSG ratio, to values of 10: 1 and even 1: 1, is an indicator of oxidative stress (Hassan and Fridovich, 1980, Zitka et al., 2012).

The reasoning for selecting 2 and 24 h as exposure times was based on the possibility of observing the biologic effects at both very early times and after a long enough exposure time to allow the initiation of key cellular responses.

ROS can promote the production of inflammatory factors such as RANTES, VEGF and EGF (Ushio-Fukai, 2007, Fay et al., 2006). It has been suggested that inflammatory cytokine responses play important roles in various pulmonary diseases such as asthma, chronic bronchitis (Matera et al., 2010). This study demonstrates that both 4-OPA and IPOH [1.5 or 31.2 μM] can effectively increase VEGF levels in A549 which appear to be dependent on the up-regulation of ROS generation. ROS-induced VEGF expression does not appear to be specific only to tumour cells but also to other type of cells (Perrot-Applanat and Di Benedetto, 2012). In fact, high VEGF levels were determined in bronchial epithelial 16HBE14o-cells exposed to both 4-OPA and IPOH [1.5 or 31.2 μM].

## 5 Conclusion

The present study provides a novel insight into the potential harmful capacity of the three common ozone-limonene reaction products: 4-OPA, IPOH and 4-AMCH on human pulmonary cells, in particular in bronchial and alveolar epithelial cell lines.

IPOH and 4-OPA treatment at non-cytotoxic concentrations induced ROS accumulation as well as alteration in the amount of a series of cytokines/chemokines (RANTES, VEGF and EGF) in alveolar (A549) and bronchial (16HBE14o-) epithelial cell lines. Of the three products analysed it was noted that 4-AMCH was the least toxic.

The results obtained, confirm the toxicity of the selected limonene-oxidation reaction products in human pulmonary cell lines and demonstrate the induction of different toxic effects related also to the specific cell type.

The experimental evidence of this study encourages further investigation of the selected chemicals which should be tested not only as single compounds but also as complex mixtures that might simulate the real environment (e.g. in presence of strong oxidants such as ozone). Furthermore, the determination of their toxicological effects, by using different exposure approaches (e.g. exposure of cells at the air-liquid interface), will allow the assessment of their toxic effect as inhalable substances.

## Disclaimer

The content of this work reflects the views of the authors and does not necessarily represent an official position of the European Commission.

## References

[bib1] Ahner B.A., Wei L., Oleson J.R., Ogura N. (2002). Glutathione and other low molecular weight thiols in marine phytoplankton under metal stress. Mar. Ecol. Prog. Ser..

[bib2] Anderson M.E. (1998). Glutathione: an overview of biosynthesis and modulation. Chem. Biol. Interact..

[bib3] Anderson S.E., Jackson L.G., Franko J., Wells J.R. (2010). Evaluation of dicarbonyls generated in a simulated indoor air environment using an in vitro exposure system. Toxicol. Sci..

[bib4] Anderson S.E., Franko J., Jackson L.G., Wells J.R., Ham J.E., Meade B.J. (2012). Irritancy and allergic responses induced by exposure to the indoor air chemical 4-oxopentanal. Toxicol. Sci..

[bib5] Anderson S.E., Khurshid S.S., Meade B.J., Lukomska E., Wells J.R. (2013). Toxicological analysis of limonene reaction products using an in vitro exposure system. Toxicol Vitro.

[bib6] Assim A. Alfadda, Reem M. Sallam (2012). Reactive oxygen species in health and disease. J. Biomed.Biotech..

[bib7] Berg J.M., Tymoczko J.L., Stryer L. (2002).

[bib8] Biswas S.K., Rahman I. (2009). Environmental toxicity, redox signalling and lung inflammation: the role of glutathione. J. Mol. Aspects Med..

[bib9] Calogirou A., Larsen B.R., Kotzias D. (1999). Gas-phase terpene oxidation products: a review. Atmos. Environ..

[bib10] Calvert P., Yao K.S., Hamilton T.C., O'Dwyer P.J. (1998). Clinical studies of reversal of drug resistance based on glutathione. Chemico-Biol. Interact..

[bib11] Carmel-Harel O., Storz G. (2000). Roles of the glutathione- and thioredoxin- dependent reduction systems in the Escherichia coli and Saccharomyces cerevisiae responses to oxidative stress. Annu. Rev. Microbiol..

[bib12] Chan F.K.-M., Moriwaki K., De Rosa M.J. (2013). Detection of necrosis by release of lactate dehydrogenase (LDH) activity. Methods Mol. Biol..

[bib13] Curtin J.F., Donovan M., Cotter T.G. (2002). Regulation and measurement of oxidative stress in apoptosis. J. Immunol. Methods..

[bib14] Dalleau S., Baradat M., Guéraud F., Huc L. (2013). Cell death and diseases related to oxidative stress: 4-hydroxynonenal (HNE) in the balance. Cell Death Differ..

[bib15] Dixon S.J., Stockwell B.R. (2014). The role of iron and reactive oxygen species in cell death. Nat. Chem. Biol..

[bib16] Ertel A., Verghese A., Byers S.W., Ochs M., Tozeren A. (2006). Pathway-specific differences between tumor cell lines and normal and tumor tissue cells. Mol. Cancer.

[bib17] Estrela J.M., Ortega A., Obrador E. (2006). Glutathione in cancer biology and therapy. Crit. Rev. Clin. Lab. Sci..

[bib18] Fay J., Varoga D., Wruck C.J., Kurz B., Goldring M.B., Pufe T. (2006). Reactive oxygen species induce expression of vascular endothelial growth factor in chondrocytes and human articular cartilage explants. Arthritis Res. Ther..

[bib19] Forester C.D., Ham J.E., Wells J.R. (2007). Geraniol (2,6-dimethyl-2,6-octadien-8-ol) reactions with ozone and OH radical: rate constants and gas-phase products. Atmos. Environ..

[bib20] Fruekilde P., Hjorth J., Jensen N.R., Kotzias D., Larsen B. (1998). Ozonolysis at vegetation surfaces: a source of acetone, 4-oxopentanal, 6-methyl-5-hepten-2-one, and geranyl acetone in the troposphere. Atmos. Environ..

[bib21] Halliwell B. (2006). Oxidative stress and neurodegeneration: where are we now?. J. Neurochem..

[bib22] Halliwell B., Whitemann M. (2004). Measuring reactive species and oxidative damage in vivo and in cell culture: how should you do it and do the results mean?. Br. J. Pharmacol..

[bib23] Hassan H.M., Fridovich I. (1980). Mechanism of the antibiotic action pyocyanine. J. Bacteriol..

[bib24] Heijink I.H., Brandenburg S.M., Noordhoek J.A., Postma D.S., Slebos D.-J., van Oosterhout A.J.M. (2010). Characterisation of cell adhesion in airway epithelial cell types using electric cell–substrate impedance sensing. ERJ.

[bib25] Hirst S.J., Barnes P.J., Twort C.H.L. (1992). Quantifying proliferation of cultured human and rabbit airway smooth muscle cells in response to serum and platelet-derived growth factor. Am. J. Respir. Cell Mol. Biol..

[bib26] Klaunig J.E., Kamendulis L.M., Hoceva B.A. (2010). Oxidative stress and oxidative damage in carcinogenesis. Toxicol. Pathol..

[bib27] Lee S., Kang H.-G., Choi J.E., Lee J.H., Kang H.J., Baek S.A., Lee E., Seok Y., Lee W.K., Lee S.Y., Yoo S.S., Lee J., Cha S.-I., Kim C.H., Cho S., Park J.Y. (2016). The different effect of VEGF polymorphisms on the prognosis of non-small cell lung cancer according to tumor histology. J. Korean Med. Sci..

[bib28] Lieber M., Smith B., Szakal A., Nelson-Rees W., Todaro G. (1976). A continuous tumor-cell line from a human lung carcinoma with properties of type II alveolar epithelial cells. Int. J. Cancer.

[bib29] Lipsa D., Cacho C., Leva P., Barrero-Moreno J., Aguar P. (2015). Development of a HPLC-UV method for the simultaneous determination of intracellular glutathione species in human cells. J. Anal. Bioanal. Tech..

[bib30] Lipsa D., Leva P., Barrero-Moreno J., Coelhan M. (2016). Inflammatory effects induced by selected limonene oxidation products: 4-OPA, IPOH, 4-AMCH in human bronchial (16HBE14o-) and alveolar (A549) epithelial cell lines. Toxicol. Lett..

[bib31] Matera M.G., Calzetta L., Cazzola M. (2010). TNF-alpha inhibitors in asthma and COPD: we must not throw the baby out with the bath water. Pulm. Pharmacol. Ther..

[bib32] Nazaroff W.W., Weschler C.J. (2004). Cleaning products and air fresheners. Exposure to primary and secondary pollutants. Atmos. Environ..

[bib33] Nørgaard A.W., Kudal J.D., Kofoed-Sørensen V., Koponen I.K., Wolkoff P. (2014). Ozone-initiated VOC and particle emissions from a cleaning agent and an air freshener: risk assessment of acute airway effects. Environ. Int..

[bib34] Owen J.B., Butterfield D.A. (2010). Measurement of oxidized/reduced glutathione ratio. Methods Mol. Biol..

[bib35] Perrot-Applanat M., Di Benedetto M. (2012). Autocrine functions of VEGF in breast tumor cells Adhesion, survival, migration and invasion. Cell Adh Migr..

[bib36] Poljsak B., Šuput D., Milisav I. (2013). Achieving the balance between ROS and antioxidants: when to use the synthetic antioxidants. Oxidative Med. Cell. Longev..

[bib37] Pope C.A., Burnett R.T., Thurston G.D., Thun M.J., Calle E.E., Krewski D., Godleski J.J. (2004). Cardiovascular mortality and long-term exposure to particulate air pollution: epidemiological evidence of general pathophysiological pathways of disease. Circulation.

[bib38] Rahman I., MacNee W. (2000). Regulation of redox glutathione levels and gene transcription in lung inflammation: therapeutic approaches. Free Rad. Biol. Med..

[bib39] Robinson J.P., Bruner L.H., Bassoe C.F., Hudson J.L., Ward P.A., Phan S.H. (1988). Measurement of intracellular fluorescence of human monocytes relative to oxidative metabolism. J. Leukoc. Biol..

[bib40] Robinson J.P., Babcock G.F. (1998). Oxygen and nitrogen reactive metabolites and phagocytic cells. Phagocyte Funct. A Guide Res. Clin. Eval..

[bib41] Rossignol S., Chiappini L., Perraudin E., Rio C., Fable S., Valorso R., Doussin J.F. (2012). Development of a parallel sampling and analysis method for the elucidation of gas/particle partitioning of oxygenated semi-volatile organics: a limonene ozonolysis. Atmos. Meas. Tech..

[bib42] Rossignol S., Rio C., Ustache A., Fable S., Nicolle J., Mome A., D'Anna B., Nicolas M., Leoz E., Chiappini L. (2013). The use of housecleaning product in an indoor environment leading to oxygenated polar compounds and SOA formation: gas and particulate phase chemical characterization. Atmos. Environ..

[bib43] Russo A., DeGraff W., Friedman N., Mitchell J.B. (1986). Selective modulation of glutathione levels in human normal versus tumor cells and subsequent differential response to chemotherapy drugs. Cancer Res..

[bib44] Sarwar G., Olson D.A., Corsi R.L., Weschler C.J. (2004). Indoor fine particles: the role of terpene emissions from consumer products. J. Air Waste Manage Assoc..

[bib45] Schmiedebergs's Naunyn (1985). Archives of pharmacology. Dtsch. Pharmakol. Ges..

[bib46] Serru V., Baudin B., Ziegler F., David J.-P., Cals M.-J., Vaubourdolle M., Mario N. (2001). Quantification of reduced and oxidized glutathione in whole blood samples by capillary electrophoresis. Clin. Chem..

[bib47] Sozzani S., Bosisio D., Mantovani A., Ghezzi P. (2005). Linking stress, oxidation and the chemokine system. Eur. J. Immunol..

[bib48] Spadaro A., Ronsisvalle G., Pappalardo M. (2011). Rapid analysis of glutathione in human prostate cancer cells (DU145) and human lung adenocarcinoma cells (A549) by HPLC with electrochemical detection. J. Pharm. Sci. Res..

[bib49] Steinemann A.C. (2009). Fragranced consumer products and undisclosed ingredients. Environ. Impact Assess. Rev..

[bib50] Sunil V.R., Laumbach R.J., Patel K.J., Turpin B.J., Lim H.-J., Kipen H.M., Laskin J.D., Laskin D.L. (2007). Pulmonary effects of inhaled limonene ozone reaction products in elderly rats. Toxicol. Appl. Pharmacol..

[bib51] Trachootham D., Lu W., Ogasawara M.A., Rivera-Del Valle N., Huang P. (2008). Redox regulation of cell survival. Antioxid. Redox Signal..

[bib52] Traverso N., Ricciarelli R., Nitti M., Marengo B., Furfaro A.L., Pronzato M.A., Marinari U.M., Domenicotti C. (2013). Role of glutathione in cancer progression and chemoresistance. Oxidative Med. Cell. Longev..

[bib53] Ushio-Fukai M. (2007). VEGF signaling through NADPH oxidase-derived ROS. Antioxid. Redox Signal.

[bib54] Uzoigwe J.C., Prum T., Bresnahan E., Garelnabi M. (2013). The emerging role of outdoor and indoor air pollution in cardiovascular disease. N. Am. J. Med. Sci..

[bib55] Vareille M., Kieninger E., Edwards M.R., Regamey N. (2011). The airway epithelium: soldier in the fight against respiratory viruses. Clin. Microbiol. Rev..

[bib56] Vignola A.M., Chanez P., Chiappara G., Merendino A., Pace E., Rizzo A., la Rocca A.M., Bellia V., Bonsignore G., Bousquet J. (1997). Transforming growth factor-β expression in mucosal biopsies in asthma and chronic bronchitis. Am. J. Respir. Crit. Care Med..

[bib57] Wang X., Luo F., Zhao H. (2014). Paraquat-induced reactive oxygen species inhibit neutrophil apoptosis via a p38 MAPK/NF-kb–IL-6/TNF-a positive-Feedback circuit. PLoS ONE.

[bib58] Wells J.R. (2005). Gas-phase chemistry of alpha-terpineol with ozone and OH radical: rate constants and products. Environ. Sci. Technol..

[bib59] Wolkoff P., Wilkins C.K., Clausen P.A., Nielsen G.D. (2006). Organic compounds in office environment – sensory irritation, odor, measurements, and the role of reactive chemistry. Indoor Air.

[bib60] Wolkoff P., Clausen P.A., Larsen K., Hammer M., Larsen S.T., Nielsen G.D. (2008). Acute airway effects of ozone-initiated d-limonene chemistry: importance of gaseous products. Toxicol. Lett..

[bib61] Wolkoff P., Larsen S.T., Hammer M., Kofoed-Sørensen V., Clausen P.A., Nielsen G.D. (2013). Human reference values for acute airway effects of five common ozone-initiated terpene reaction products in indoor air. Toxicol. Lett..

[bib62] Zietkowski Z., Tomasiak M.M., Skiepko R., Bodzenta-Lukaszyk A. (2008). RANTES in exhaled breath condensate of stable and unstable asthma patients. Resp. Med..

[bib63] Zitka O., Skalickova S., Gumulec J., Masarik M., Adam V., Hubalek J., Trnkova L., Kruseova J., Eckschlager T., Kizek R. (2012). Redox status expressed as GSH: GSSG ratio as a marker for oxidative stress in paediatric tumour patients. Oncol. Lett..

